# Oral Isotretinoin Therapy in Recalcitrant Molluscum Contagiosum in an Immunocompromised Patient

**DOI:** 10.1155/2021/5529382

**Published:** 2021-06-14

**Authors:** Vikash Paudel, Deepa Chudal

**Affiliations:** ^1^Department of Dermatology and Venereology, National Medical College, Birgunj, Nepal; ^2^Nepal Police Hospital, Kathmandu, Nepal

## Abstract

Molluscum contagiosum is a viral infection caused by the Poxvirus characterized by multiple umbilicated papules. It is common in children and can be present at any body site. Severe molluscum is common in immunocompromised patients. We report a 20-year-old HIV-positive individual with widespread molluscum contagiosum, recalcitrant to topical therapy, under antiretroviral therapy, who was treated with oral isotretinoin and had a dramatic outcome. Although studies are needed to confirm the effectiveness of oral isotretinoin therapy in molluscum contagiosum, its easy availability, cost, and excellent safety profile appear to offer a promising therapeutic option.

## 1. Introduction

Molluscum contagiosum (MC) is a viral infection caused by a DNA virus, and the transmission is by direct contact. It is common in children and immunosuppressed adults [1]. Although topical retinoids have been used in the treatment of MC, their systemic use in MC is not reported in the literature. Thus, we report a rare case of recalcitrant MC where the efficacy of oral isotretinoin for the treatment of severe and recalcitrant MC in an HIV patient is demonstrated.

## 2. Case Report

A 20-year-old male patient presented to the outpatient clinic with an eruption of widespread skin-colored, dome-shaped lesions over his face and neck for one year. The lesions were asymptomatic but gradually increasing in size and number. A routine history revealed that the patient was HIV positive along with both of his parents. His CD4 + count was 82 cells/mm3 (normal > 500/mm3). He had the signs and symptoms of neither HIV nor the opportunistic infections. Besides, his medical and surgical history was also insignificant. He was taking standard antiretroviral therapy (ART) regimen regularly for the last five years, according to the national guidelines of Nepal. The ART used was tenofovir (300 mg), lamivudine (300 mg), and efavirenz (600 mg). For those eruptions, he was prescribed multiple topical medications (salicylic acid 5%, retinoic acid 0.05%, and other local remedies made from plant extract) and oral medications (not documented). However, there was no significant improvement of the lesions. No systemic modalities of retinoid or other antiviral medication were used previously.

**Figure 1:**
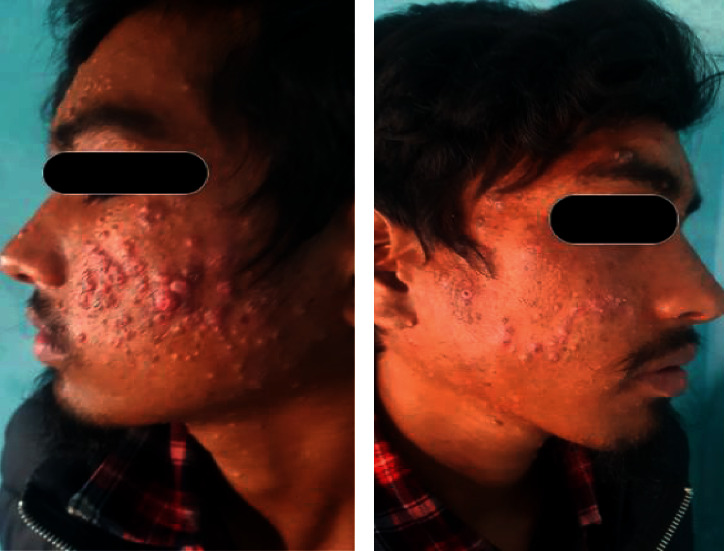
(a, b) MC lesions before oral isotretinoin.

**Figure 2:**
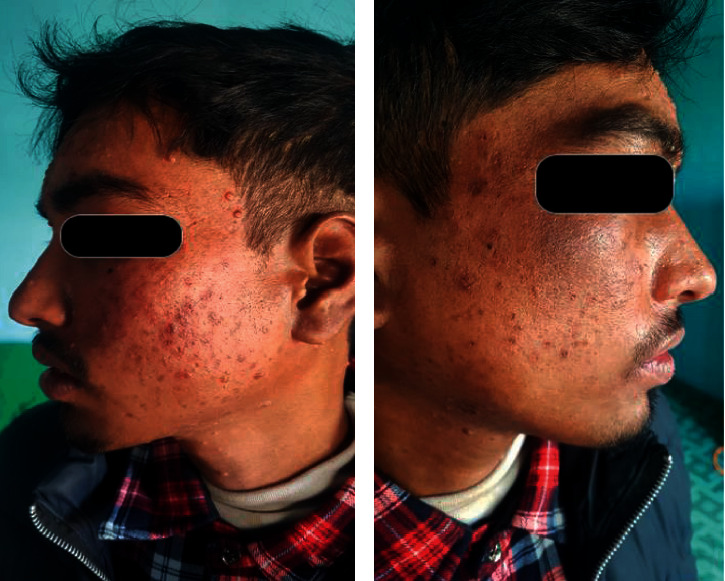
(a, b) Resolution of MC after one month of therapy with oral isotretinoin.

On examination, several lesions were widespread over the eyelids, forehead, cheeks, nose, and neck, sparing the trunk and extremities (Figures 1(a) and 1(b)). They were pearly in color, umbilicated, dome-shaped papules which were discrete to coalescing, with a size ranging from a few millimeters to one centimeter. We made our clinical diagnosis as molluscum contagiosum in an immunocompromised patient and confirmed it after extirpation with a needle to find molluscae bodies in some of the skin lesions. Skin biopsy was not considered an option for diagnosis as a clinical diagnosis was sufficient in such cases. Dermoscopy might have assisted us with the confirmative diagnosis but was not performed due to its unavailability. Due to the widespread location in the face and neck, recalcitrant nature, unavailability of other proven therapies (imiquimod gel, ingnelol gel, interferon, and cidopovir), and possibilities of side effects associated with systemic drugs, we decided to treat the patient with retinoid derivatives but systemically, i.e., oral isotretinoin 0.5 mg/kg. Isotretinoin was used as an option because of its property of being a systemic retinoid with intracellular conversion into tretinoin and its effects on cellular proliferation and differentiation. After discussing its possible side effects and measures to prevent them, isotretinoin (20 mg twice daily) was prescribed for a month to see the possible outcome. After one month of follow-up, there was subjective (both the patient and physician) and objective improvement in the appearance and count of the lesions with almost no lesions in the right half of the face and few lesions in the left half of the face (Figures 2(a) and 2(b)). The patient had no adverse effects of oral isotretinoin use except dry lips which were managed using petrolatum jelly. The remission was maintained even after two months of follow-up.

## 3. Discussion

Molluscum contagiosum is a cutaneous viral infection that is caused by a DNA virus that replicates only in human epidermal keratinocytes [1]. The virus is assumed to be the only existing poxvirus that specifically affects human beings. MC virus has 4 different genotypes: MCV 1, MCV 2, MCV 3, and MCV 4. It is transmitted by direct contact with infected skin, sexual, nonsexual, or by autoinoculation. Additionally, it can be transmitted by contaminated fomites.[2]. It usually presents as single or multiple, spherical, shiny, pearly white papules that classically have a central dimple. In immunocompetent patients, infection is self-limited with spontaneous resolution. Risk factors for infection in adult patients include sexual transmission and severe immunosuppression. Extragenital molluscum contagiosum in adults occurs almost exclusively in HIV-infected or immunocompromised ones. People with HIV infection are more prone to molluscum contagiosum; prevalence in this population has been reported up to 20% [3].

In immunocompetent individuals, MC is self-limiting with spontaneous resolution. Resolution of the lesions could be accelerated by its destruction and production of inflammatory responses. Many treatment options such as doing nothing (natural resolution), physical destruction (i.e., cryotherapy, extraction, and curettage and pricking with a needle), and topical agents (tretinoin, tazarotene, salicylic acid, imiquimod, potassium hydroxide, podophyllin, phenol, and contact immunotherapies) are discussed in the literature. Few systemic treatment modalities such as antivirals (cidofovir and interferon) or cimetidine have been suggested as a possible treatment for their antiviral and systemic immunomodulatory effects. Besides, interferon alfa is a proinflammatory cytokine that is used in the treatment of MC in immunosuppressed patients with severe or refractory disease and can be administered subcutaneously or intralesionally [4]. No therapy is universally effective in MC in HIV patients except the initiation of ART therapy [5]. Because the unavailability of such systemic therapies and the patient being already on an ART regimen, oral isotretinoin was tried as an alternative due to its effects in the keratinocytes microenvironment.

Retinoids have been used in dermatology for many decades; indications for their use include psoriasis, acne, and disorders of keratinization. Their mechanism is attributed to the ability to control abnormal growth and differentiation of keratinocytes. Isotretinoin has remarkable efficacy on keratinocytes by influencing cell cycle progression, cellular differentiation, cell survival, and apoptosis [6]. It results in a significant reduction in sebum production and has anti-inflammatory properties. Study data suggest that all-trans-retinoic acid may be the active intracellular form of isotretinoin after isomerization which induces apoptosis in cells cultured from different human cells. Although oral isotretinoin has no direct antimicrobial action, it alters the microenvironment of keratinocytes leading to an unfavorable milieu for the poxvirus to survive which could be one possible mechanism of its efficacy in MC [7]. The role of isotretinoin in our condition might also be explained by its efficacy from the Fox O1 hypothesis leading to apoptosis of virally infected cells and resolution of MC [8].

When evaluating the response rate of any treatment modality, the possibility of spontaneous resolution should be considered as in other cases of MC. Notably, the patient had exhibited long durations of MC and had failed to respond to the known treatment modalities. Hence, the results were less likely to be influenced by the effect of spontaneous remission, and isotretinoin could be a newer modality of treatment in recalcitrant MC.

## 4. Conclusions

Although topical retinoic acids have been used in the treatment of molluscum, oral isotretinoin could be promising in widespread and recalcitrant MC. Indeed, randomized controlled studies are needed to confirm the effectiveness of this treatment, but its excellent safety profile appears to offer an attractive therapeutic option.

## Data Availability

No data were used in this study.
